# Bicarbonate and Serum Lab Markers as Predictors of Mortality in the Trauma Patient

**DOI:** 10.5811/westjem.18363

**Published:** 2024-05-20

**Authors:** Matthew M. Talbott, Angela N. Waguespack, Peyton A. Armstrong, John W. Davis, Krishna K. Paul, Shania M. Williams, Georgiy Golovko, Joshua Person, Dietrich Jehle

**Affiliations:** * University of Texas, Medical Branch, Department of Emergency Medicine, Galveston, Texas; † University of Texas, Medical Branch, Department of Pharmacology, Galveston, Texas; ‡ University of Texas, Medical Branch, Department of Surgery, Galveston, Texas

## Abstract

**Introduction:**

Severe trauma-induced blood loss can lead to metabolic acidosis, shock, and death. Identification of abnormalities in the bicarbonate and serum markers may be seen before frank changes in vital signs in the hemorrhaging trauma patient, allowing for earlier lifesaving interventions. In this study the author aimed to evaluate the usefulness of serum bicarbonate and other lab markers as predictors of mortality in trauma patients within 30 days after injury.

**Methods:**

This retrospective, propensity-matched cohort study used the TriNetX database, covering approximately 92 million patients from 55 healthcare organizations in the United States, including 3.8 million trauma patients in the last two decades. Trauma patients were included if they had lab measurements available the day of the event. The analysis focused on mortality within 30 days post-trauma in comparison to measured lab markers. Cohorts were formed based on ranges of bicarbonate, lactate, and base excess levels.

**Results:**

Before propensity score matching, a total of 1,275,363 trauma patients with same-day bicarbonate, lactate, or base excess labs were identified. A significant difference in mortality was found across various serum bicarbonate lab ranges compared to the standard range of 21–27 milliequivalents per liter (mEq/L), post-propensity score matching. The relative risk of death was 6.806 for bicarbonate ≤5 mEq/L; 8.651 for 6–10; 6.746 for 11–15; 2.822 for 16–20; and 1.015 for bicarbonate ≥28. Serum lactate also displayed significant mortality outcomes when compared to a normal level of ≤2 millimoles per liter. Base excess showed similar significant correlation at different values compared to a normal base excess of −2 to 2 mEq/L.

**Conclusion:**

This study, approximately 100 times larger than prior studies, associated lower bicarbonate levels with increased mortality in the trauma patient. While lactate and base excess offer prognostic value, lower bicarbonate values have a higher relative risk of death. The greater predictive value of bicarbonate and accessibility during resuscitations suggests that it may be the superior prognostic marker in trauma.

Population Health Research Capsule What do we already know about this issue?

*Base excess and lactate levels are strong predictors of mortality in trauma patients. Bicarbonate levels, while related, are a more convenient and possibly superior alternative.*

What was the research question?

*Is serum bicarbonate level the superior prognostic marker in trauma?*

What was the major finding of the study?

*Lower bicarbonate values (ranges from 20 to ≤5) were strongly associated with increasing risks of mortality (P < 0.001).*

How does this improve population health?

*This study suggests that serum bicarbonate is superior to lactate and base excess in predicting trauma mortality.*

## INTRODUCTION

Trauma is a leading cause of mortality among individuals <45 years of age and the elderly.^1^ Hemorrhage-induced hypovolemia can result in inadequate oxygen delivery to tissues, leading to metabolic acidosis. Early identification of shock in trauma patients is crucial as it can facilitate interventions that mitigate ongoing tissue damage and improve survival. Metabolic acidosis is a significant prognostic indicator for the severity of hemorrhage in trauma cases.^2^

Both vital signs and laboratory measurements provide essential guidance for improving the outcomes of resuscitation in critically ill patients.^3^^,^^4^ Several studies have attempted to predict mortality in major trauma patients using acid-base measures.^4^^,^^5^ Many of these studies have revealed that serum lactate is a reliable predictor of mortality in severely injured patients^4^^,^^6^^,^^7^ and may even outperform arterial base deficit as a predictive tool.^8^^,^^9^ Additionally, some smaller studies have indicated that both arterial and venous bicarbonate values can effectively predict mortality in critical care settings.^2^^,^^7^^,^^8^ Serum bicarbonate and base deficit have been found to be approximately equivalent in their predictive capacity in other studies.^7^ Given that lactic acid measurements and arterial base deficit may not be immediately available at the time of a patient’s initial presentation,^9^ further exploration of the predictive value of bicarbonate measures becomes critical.

The author’s primary objective in this study was to assess the utility of serum bicarbonate and other acid-base markers in the evaluation of trauma patients who present to the emergency department. This evaluation was conducted using a comprehensive retrospective healthcare database. The specific aim was to determine the predictive value of serum bicarbonate and other laboratory markers in forecasting mortality in trauma patients up to 30 days after their injury.

## METHODS

### Design

This was a retrospective, propensity-matched cohort study using the TriNetX database. TriNetX is a large, global research network that provides de-identified medical information. The United States Collaborative Network in TriNetx represents approximately 92 million patients and 55 large healthcare organizations (HCO) within the US. The network accesses electronic health record information that includes diagnoses, procedures, medications, and laboratory data.^10^ The TriNetX database includes admitted and discharged patients, as well as office visits, in contrast to the National Trauma Data Bank, which only includes admitted patients. For this analysis, the author included health records over a 20-year period from November 2, 2002–November 2, 2022.

### Participants

Cohort exposure was defined as serum bicarbonate level at baseline (bicarbonate [moles/volume] in serum, plasma, or blood, TMX: 9021) with any trauma-related International Classification of Diseases, Rev 9 or 10 (ICD-9 or ICD-10 code (ICD10CM: T14; ICD-9 xxx). Approximately 90% of the bicarbonate values were obtained from venous samples, with the remaining 10% from arterial samples. Persons <18 years old or without lab values available from the day of event were excluded. The measured outcome was death within 30 days of the indexed traumatic event. At least 94% of the HCOs in the TriNetX database are linked to the US National Death Registries. Patients were excluded if the indexed traumatic event occurred greater than 20 years from date of analysis.

The control cohort was defined as all persons with trauma who had a normal bicarbonate level (21–27 milliequivalents per liter [mEq/L]) at baseline. There are variable definitions of the normal ranges for bicarbonate, lactate, and base excess (BE) in the literature; therefore, round cutoffs were chosen for interpretation purposes. The control cohort was compared to other cohorts with a varying range of bicarbonate values. These ranges of bicarbonate included ≤5, 6–10, 11–15, 16–20, and ≥28 mEq/L. For comparative effectiveness analysis, the author then repeated the analysis for lactic acid and BE as they have been studied in previous research. The control cohort was a normal lactic acid of ≤2.0 millimoles per liter (mmol/L). The control cohort was compared to lactic acid levels at varying ranges, at 2 mmol/L increments. For BE, our control group was a normal BE, between −2.0–2.0 mmol/L. The BE control group was matched against cohorts at varying ranges of BE, at 2 mmol/L increments. All BE measurements were obtained from arterial samples.

### Statistical Analysis

To control for potential confounding demographic factors, the propensity matching tool in TriNetX was used. Using these matches, the researcher can estimate the difference between both groups without the influence of the confounding variables.^10^

The cohort was analyzed descriptively using univariate and bivariate frequencies with chi square and t-testing to assess differences. All eligible persons in the cohort were analyzed using both binary event estimation with risk ratios (RR), 95% confidence intervals (CI), and probability values. Using the TriNetX database, the author employed a 1:1 propensity match using logistic regression for age, gender, race, and ethnicity for maximum generalization to the US population. Greedy nearest-neighbor matching was used with a tolerance of 0.1 and difference between propensity scores ≤0.1. Comparisons were made between cohort before and after propensity matching. This study methodology has been previously validated in the TriNetX platform.^11^ Statistical significance was set at a two-sided alpha <0.05. TriNetX provides data that has been de-identified; therefore, IRB review was not required for this study.^12^ The final analysis was run on November 2, 2022.

## RESULTS

There were 92,529,034 patients in the US Collaborative Network from 55 HCOs within the TriNetX database. There were a total of 3,892,737 patients with a traumatic mechanism, and 28,967,134 patients with serum bicarbonate lab values. A total of 1,275,363 trauma patients were identified before propensity matching, who had received a bicarbonate, lactate, or BE lab on the same day of a trauma incident (Table 1).

**Table 1.** Cohort demographics.

Demographics	Mean	±SD
Age	55	±22
	Percentage	
Gender		
Male	53%	
Female	45%	
Unknown	2%	
Ethnicity		
Not Hispanic or Latino	76%	
Hispanic or Latino	8%	
Unknown Ethnicity	16%	
Race		
White	68%	
Black	17%	
American Indian or Alaskan	1%	
Asian	1%	
Native Hawaiian or other Pacific Islander	0%	
Unknown race	12%	
Other race	1%	
Common comorbidities		
Hypertensive diseases	49%	
Other forms of heart diseases	42%	
Other anxiety disorders	30%	
Overweight and obesity	24%	
Diabetes mellitus	23%	

### Bicarbonate

For the bicarbonate group, a total of 1,275,363 patients were identified before propensity matching: 814,895 patients with bicarbonate 21–27 mEq/L (control); 2,643 with bicarbonate ≤5 mEq/L; 5,949 with bicarbonate 6–10 mEq/L; 25,882 with bicarbonate 11–15 mEq/L; 160,886 with bicarbonate 16–20 mEq/L; and 265,108 with bicarbonate ≥28 mEq/L. After propensity matching, patients with bicarbonate 6–10 mEq/L had the highest risk of death when compared to control at 25.9% vs 3.0% (RR 8.65, 95% CI 7.432–10.070, *P* < 0.001), and decreased as bicarbonate decreased, with the lowest being ≥ 28 mEq/L at 3.5% vs 3.4% (RR: 1.015, 95% CI 0.986–1.044, *P* = 0.32) which was not statistically significant. When compared to control, patients with bicarbonate ≤5 mEq/L (19.8%, RR 6.8) had similar risks of mortality as 11–15 mEq/L (20.0%, RR 6.9). Mortality followed a similar trend before propensity matching (Table 2).

**Table 2.** 30-day mortality when compared to normal serum bicarbonate (21–27 milliequivalents per liter).

	Before propensity score matching			After propensity score matching		
Serum bicarbonate (mEq/L)	Mortality	RR (95% CI)	*P*-value	Mortality	RR (95% CI)	*P*-value
≤5 21–27	19.8% 2.9%	6.8 (6.3, 7.4)	<0.001	19.8% 2.9%	6.8 (5.3, 8.6)	<0.001
6–10 21–27	25.9% 2.9%	8.9 (8.5, 9.4)	<0.001	25.9% 3.0%	8.7 (7.4, 10.1)	<0.001
11–15 21–27	20.0% 2.9%	6.9 (6.7, 7.1)	<0.001	20.0% 3.0%	6.7 (6.3, 7.3)	<0.001
16–20 21–27	8.2% 2.9%	2.8 (2.8, 2.9)	<0.001	8.2% 2.9%	2.8 (2.7, 2.9)	<0.001
≥28 21–27	3.5% 2.9%	1.2 (1.2, 1.2)	<0.001	3.5% 3.4%	1.0 (1.0, 1.0)	0.32

*CI*, confidence interval; *RR*, risk ratio; *mEq/L*, milliequivalents per liter.

### Lactate

For the lactate group, a total of 326,562 patients were identified before propensity matching: 195,457 patients with lactate ≤ 2 moles/volume (control); 86,989 with lactate 2.01–4 moles/volume; 23,120 with lactate 4.01–6 moles/volume 9,540 with lactate 6.01–8 moles/volume, and 11,456 with ≥8.01 moles/volume. After propensity matching, mortality was shown to increase as lactate levels increased. When compared to the control, the lowest RRs for mortality were within the 2.01–4 moles/volume range at 9.2% vs 5.1% (RR 1.814, 95% CI 1.751–1.880, *P* < 0.001), and reached the highest risks when ≥8.01 moles/volume at 31.7% vs 4.9% (RR 6.420, 95% CI 5.895–6.991, *P* < 0.001). Mortality followed a similar trend before propensity matching (Table 3).

**Table 3.** 30-day mortality when compared to normal lactate (≤2 moles/volume) before propensity score matching.

	Before propensity score matching			After propensity score matching		
Lactate (moles/volume)	Mortality	RR (95% CI)	*P*-value	Mortality	RR (95% CI)	*P*-value
2.01 – 4 ≤2	9.2% 5.2%	1.8 (1.7, 1.8)	<0.001	9.2% 5.1%	1.8 (1.8, 1.9)	<0.001
4.01 – 6 ≤2	17.4% 5.2%	3.3 (3.2, 3.4)	<0.001	17.4% 5.0%	3.5 (3.3, 3.4)	<0.001
6.01 – 8 ≤2	26.2% 5.2%	5.0 (4.8, 5.2)	<0.001	26.2% 5.0%	5.2 (4.7, 5.7)	<0.001
≥8.01 ≤2	31.7% 5.2%	6.0 (5.8, 6.2)	<0.001	31.7% 4.9%	6.4 (5.9, 7.0)	<0.001

*CI*, confidence interval; *RR*, risk ratio.

### Base Excess

For the BE group, a total of 34,717 patients were identified before propensity matching: 19,387 patients with BE −2 to2 mmol/L (control); 5,161 with BE −4 to −2.01 mmol/L; 3,525 with BE −6 to −4.01 mmol/L; 2,359 with BE −8 to −6.01 mmol/L; 1,585 with BE −10 to −8.01 mmol/L; and 2,700 with ≤ −10.01 mmol/L. After propensity matching, mortality was shown to increase as BE levels decreased. When compared to the control range, BE −4 to −2.01 mmol/L showed the lowest mortality risks at 6.3% vs 4.8% (RR 1.308, 95% CI 1.113–1.538, *P* = 0.001), which increased to the highest point when BE was ≤ −10.01 mmol/L at 21.8% vs 5.1% (RR 4.309, 95% CI 3.601–5.156, *P* < 0.001). Mortality followed a similar trend before propensity matching, although RR was somewhat lower (Table 4).

**Table 4.** 30-day mortality when compared to normal base excess (−2 to 2 millimoles per liter).

	Before propensity score matching			After propensity score matching		
Base excess (mmol/L)	Mortality	RR (95% CI)	*P*-value	Mortality	RR (95% CI)	*P*-value
−4 to −2.01 −2 to 2	6.3% 5.9%	1.1 (0.9, 1.2)	0.30	6.3% 4.8%	1.3 (1.1, 1.5)	0.001
−6 to −4.01 −2 to 2	8.2% 5.9%	1.4 (1.2, 1.6)	<0.001	8.2% 4.8%	1.7 (1.4, 2.0)	<0.001
−8 to −6.01 −2 to 2	11.6% 5.9%	2.0 (1.7, 2.2)	<0.001	11.6% 4.7%	2.5 (2.0, 3.0)	<0.001
−10 to −8.01 −2 to 2	14.7% 5.9%	2.5 (2.2, 2.9)	<0.001	14.7% 4.6%	3.2 (2.5, 4.1)	<0.001
≤ −10.01 −2 to 2	21.8% 5.9%	3.7 (3.4, 4.1)	<0.001	21.8% 5.1%	4.3 (3.6, 5.2)	<0.001

*CI*, confidence interval; *RR*, risk ratio; *mmol/L*, millimoles per liter.

## DISCUSSION

In this study the author explored the possibility that serum bicarbonate was a more powerful predictor of mortality at 30 days following a presentation for trauma in the emergency department than lactate or BE. While arterial base deficit likewise demonstrated predictive utility, as in previous studies, this measure required an arterial blood sample.^4^^,^^8^ This novel finding suggests serum bicarbonate can provide a rapid, easily obtainable assessment of a trauma patient at initial presentation. Lower serum bicarbonate levels were associated with a greater risk of mortality at 30 days than those with normal range bicarbonate levels. Many previous studies have demonstrated a high degree of correlation between serum lactate and serum bicarbonate in the setting of trauma,^8^ but none have quantitatively defined that risk in such a dataset. This study is approximately 75 times larger than any other study in the literature that has looked at the relationship between serum bicarbonate levels and 30-day mortality in patients presenting for trauma.

Shane et al showed that a lower serum bicarbonate level is associated with a significant increase in mortality, which is in line with our study. Their study had a smaller sample population of 93.^4^ In the Shane study, they proposed that the difference in bicarbonate levels in those who survived was significantly different vs those who expired, especially within 24 hours of trauma sustained. While they also suggested that the underdeveloped area of Uganda and small sample size may have played a role in the data collected, the venous levels of bicarbonate do show that those within a normal range had a statistically significant survival advantage.

Hussein et al performed a small study that showed elevated lactic acid levels were associated with an increase in mortality. They also demonstrated that base deficit could predict mortality in the trauma patient. Their study is somewhat limited as it had a total of 137 patients with only 36 being trauma patients.^8^ Hussein et al also demonstrated an increase in mortality with significant differences in base deficit after 24 hours in patients in the surgical intensive care unit, although the initial base deficit was not significantly different. Furthermore, they proposed that the initial base deficit (vs the 24-hour reading) did not correlate with the lactate levels and was not a reliable predictor of mortality, except in the instance of deaths due to trauma (37 of 100 total patients) further showing that acid/base differences can be a predictor of mortality in trauma.^8^

FitzSullivan et al showed a correlation between arterial base deficit and serum bicarbonate and may be used interchangeably in trauma resuscitation. Their study had 3,102 patients with 50,311 matched laboratory datasets.^7^ FitzSullivan et al set out to draw a linear correlation between arterial base deficit and serum bicarbonate (HCO_3_) in relation to the severity of injury and death. Since the base deficit is acquired through arterial puncture, HCO_3_ could provide for a substitute marker as it is normally drawn on admission. Their data showed the predictive ability of HCO_3_ in trauma cases with regard to its comparison to base deficit in the same cases.^7^ In addition, the bicarbonate outperformed lactic acid in predicting mortality. This further shows that bicarbonate can accurately and reliably be used as a predictor of mortality in trauma patients.

Mutschler et al performed a study with 16,305 patients from a trauma registry and showed a significant correlation between worsening base deficit and mortality.^(13)^ Caputo et al found that lactate and base deficit correlated well with each other as indicators of the presence of occult shock in a group of 100 trauma patients.^(14)^ Callaway et al found that lactate and base deficit were associated with increased mortality in a group of 588 elderly trauma patients.^2^ These studies and others^2^^–^^4^^,^^7^^,^^8^^,^^13^^–^^21^ that have evaluated lab markers have a smaller patient population compared to the current study of over three million trauma patients. Because of this, the author considers his study to hold more power and predictive ability in evaluating the serum lab markers in trauma.

While this propensity matched study provides powerful, generalizable estimates of mortality risk with bicarbonate levels, the author also performed non-matched estimations as a sensitivity analysis. These estimates did not meaningfully differ from those that were propensity matched, suggesting that confounders attributable to the demographics were not meaningful in this database.

## LIMITATIONS

There are a number of limitations to this study. As with all observational studies in electronic databases, causal effects cannot be inferred. There are many reasons why a patient with trauma might present with metabolic acidosis, such as age, increased likelihood of comorbidities (ie, heart failure, chronic obstructive pulmonary disease [COPD], diabetes mellitus), underlying anemia, or later presentation to emergency services. Clinical details about each patient encounter such as Injury Severity Score (ISS), mechanism of injury, and other resuscitative variables that may affect mortality endpoint are not all captured in the database, which can limit predictability of lab results on mortality. The ISS scores, however, are typically available at discharge, and this study evaluated patients on arrival.

Propensity score matching was employed for demographics such as age, race, ethnicity, and gender; despite this, there could have been other variables that may have affected outcomes that were not adjusted for in the study. Additionally, covariates chosen for propensity matching were consistent between groups. Variables that may affect one group (ie, renal failure/COPD might affect bicarb but not lactates) were not considered. Labs were gathered on the same date as initial trauma and not specifically the first lab value. There is also a possibility that patients can belong to multiple lab-testing groups. As this study contains a large number of trauma patients, these limitations should minimally affect the data.

## CONCLUSION

Metabolic acidosis is an ominous sign in the setting of initial trauma presentation and has been long associated with increased mortality rates. In this retrospective, propensity-matched study of a large cohort of patients presenting to the emergency department with trauma, we found an increased mortality risk with lower serum venous bicarbonate measurements. The serum bicarbonate outperformed lactate and base excess with a higher risk ratio of death for lower bicarbonate values. Because of this greater prognostic value and availability, we recommend routine collection of serum bicarbonate rather than lactate or arterial base deficit at the point of presentation to guide management of the trauma patient.
